# Characterization of a recombinant pseudorabies virus expressing porcine parvovirus VP2 protein and porcine IL-6

**DOI:** 10.1186/s12985-020-1292-8

**Published:** 2020-02-03

**Authors:** Hui-Hua Zheng, Lin-Qing Wang, Peng-Fei Fu, Lan-Lan Zheng, Hong-Ying Chen, Fang Liu

**Affiliations:** 1 College of Animal Science and Veterinary Medicine, Henan Agricultural University, Zhengdong New District Longzi Lake#15, 450046 Zhengzhou, Henan Province People’s Republic of China; 2 Department of Life Science, Zhengzhou Normal University, Zhengzhou, 450044 Henan Province People’s Republic of China

**Keywords:** Porcine interleukin-6, Porcine parvovirus, Recombinant pseudorabies virus, VP2 protein, Microbiology, Immunology, Virology, Viral immunology

## Abstract

**Background:**

Porcine parvovirus (PPV) and pseudorabies virus (PRV) are the important etiological agents of swine infectious diseases, resulting in huge economic losses to the Chinese swine industry. Interleukin-6 (IL-6) has the roles to support host immune response to infections as a pleiotropic cytokine. It is essential to construct a live attenuated vaccine-based recombinant PRV that expresses PPV VP2 protein and porcine IL-6 for prevention and control of PRV and PPV.

**Methods:**

The recombinant plasmid, pGVP2-IL6, was constructed by porcine IL-6 gene substituting for EGFP gene of the PRV transfer plasmid pGVP2-EGFP containing VP2 gene of PPV. Plasmid pGVP2-IL6 was transfected into swine testicle cells pre-infected with the virus rPRV-VP2-EGFP strain through homologous recombination and plaque purification to generate a recombinant virus rPRV-VP2-IL6. The recombinant PRV was further identified by PCR and DNA sequencing, and the expression of the VP2 protein and porcine IL-6 was analyzed by reverse transcription-PCR (RT-PCR) and Western blot. The virus titer was calculated according to Reed and Muench method. The immunogenicity of the recombinant virus was preliminarily evaluated in mice by intramuscular administration twice with the rPRV-VP2-IL6 at 4-week intervals.

**Results:**

A recombinant virus rPRV-VP2-IL6 was successfully constructed and confirmed in this study. The properties of rPRV-VP2-IL6 were similar to the parental virus HB98 in terms of growth curve, morphogenesis and virus plaque sizes, and rPRV-VP2-IL6 was proliferated in different cell types. It induced specific antibodies against PPV as well as a strong increase of PPV-specific lymphocyte proliferation responses in mice immunized with rPRV-VP2-IL6, and provided partial protection against the virulent PPV challenge. rPRV-VP2-IL6 also induced a high level of neutralizing antibodies against PRV, and significantly reduced the mortality rate of (1 of 10) following virulent PRV challenge compared with the control (10 of 10).

**Conclusions:**

The recombinant rPRV-VP2-IL6 might be a potential candidate vaccine against PRV and PPV infections in pigs.

## Introduction

Porcine parvovirus (PPV) was found by Cartwright in 1967 in swine affected with reproductive failure, and a worldwide distribution was observed thereafter [1–4]. Belonging to the *Parvoviridae* family, PPV is considered to be the major causative agent of reproductive failure in pregnant sows characterized by stillbirths, mummified fetuses, early embryonic death, infertility and delayed return to estrus [4, 5] . In addition, PPV has been implicated as the causative agent of diarrhea, skin disease and arthritis in swine, and often infects swine together with porcine reproductive and respiratory syndrome virus (PRRSV), porcine circovirus 2 (PCV2) and other pathogens [6, 7]. PPV has a single-stranded negative-sense DNA genome encapsidated by a non-enveloped icosahedral particle of 25 nm in diameter that is composed of three structural proteins: VP1, VP2 and VP3. Capsid VP2 protein, one of the major structural proteins of PPV, induced PPV-neutralizing antibodies to neutralize PPV infection and played a key role in PPV diagnosis and immune prophylaxis [8–10]. Moreover, VP2 protein took part in the forming of PPV empty capsid particles by self-assemble [11, 12]. PPV inactivated oil emulsion whole virus vaccines have played an important role in PPV control. Inactivated vaccine needs to be given as multiple vaccinations, and does not prevent virus shedding even after homologous virus challenge [13]. Thus, the cost of production and laborious administration of inactivated vaccine, are limitations for their wide application in the field. Genetic engineered subunit vaccines [11, 14, 15] that could induce specific immune responses and have shown efficacy against challenge virus are under development.

Pseudorabies virus (PRV), a member of the subfamily *Alphaherpesvirinae* of the *Herpesviridae* family, is a linear DNA molecule of 143 kilobases [16]. The large genome of PRV is capable of accommodating several kilobases (kb) of foreign DNA, and stable expression of foreign genes does not affect the stability of the virus itself. The possible insertion sites include the TK, PK, gE, gI and gG genes which are not essential for viral replication [17, 18], and the inactivation or deletion of one or more of these genes leads to an attenuated phenotype while retaining the replication ability of the virus [19]. The attenuated PRV is safe for pigs of all ages, but it still maintains good immunogenicity, which can simultaneously stimulate humoral immunity and cell-mediated immunity [20, 21]. PRV has been used for the integration and expression of foreign genes as a powerful vector system [22–26], and vaccinated or wide PRV infected swine can be discriminated by a serological assay that detects antibodies against the deleted gene (e.g., gE and gG) [27]. Therefore, attenuated PRV may be a promising candidate vaccine vector for the expression and delivery of other antigens to confer protection against both pseudorabies and other animal diseases [25, 26, 28].

Interleukin-6 (IL-6) is a pleiotropic cytokine which plays an essential role in host immune response [29]. IL-6 was initially recognized as a factor to induce immunoglobulin production in activated B cells and to exhibit a wide range of biological functions in cells outside the B lymphocyte system [30]. Previous researches indicated that recombinant IL-6 could enhance protective immune responses to DNA vaccine in mice [31, 32]. Therefore, in the present study, we constructed an HB98-derived recombinant pseudorabies virus rPRV-VP2-IL6 expressing the VP2 protein and porcine IL-6, and evaluated its growth properties, chemical and physical properties, genetic stability, and immunogenicity in mice. This study will lay the foundation for a potential candidate vaccine to prevent and control both PRV and PPV infections in pigs.

## Materials and methods

### Cells, virus and plasmids

Cells used in this study including swine testicle (ST) cells, porcine small intestine epithelial cells IPEC, porcine kidney cell lines IBRS2 and PK-15 (ATCC™ CCL-33), and green monkey kidney cell line Vero were purchased from the China Institute of Veterinary Drug Control, Beijing, China, and maintained in Dulbecco’s modified Eagle medium (DMEM) (HyClone laboratory Inc., Longa, UT, USA) supplemented with 10% fetal bovine serum (FBS; GIBCO BRL, Gaithersburg, MD, USA).

**Fig. 1:**
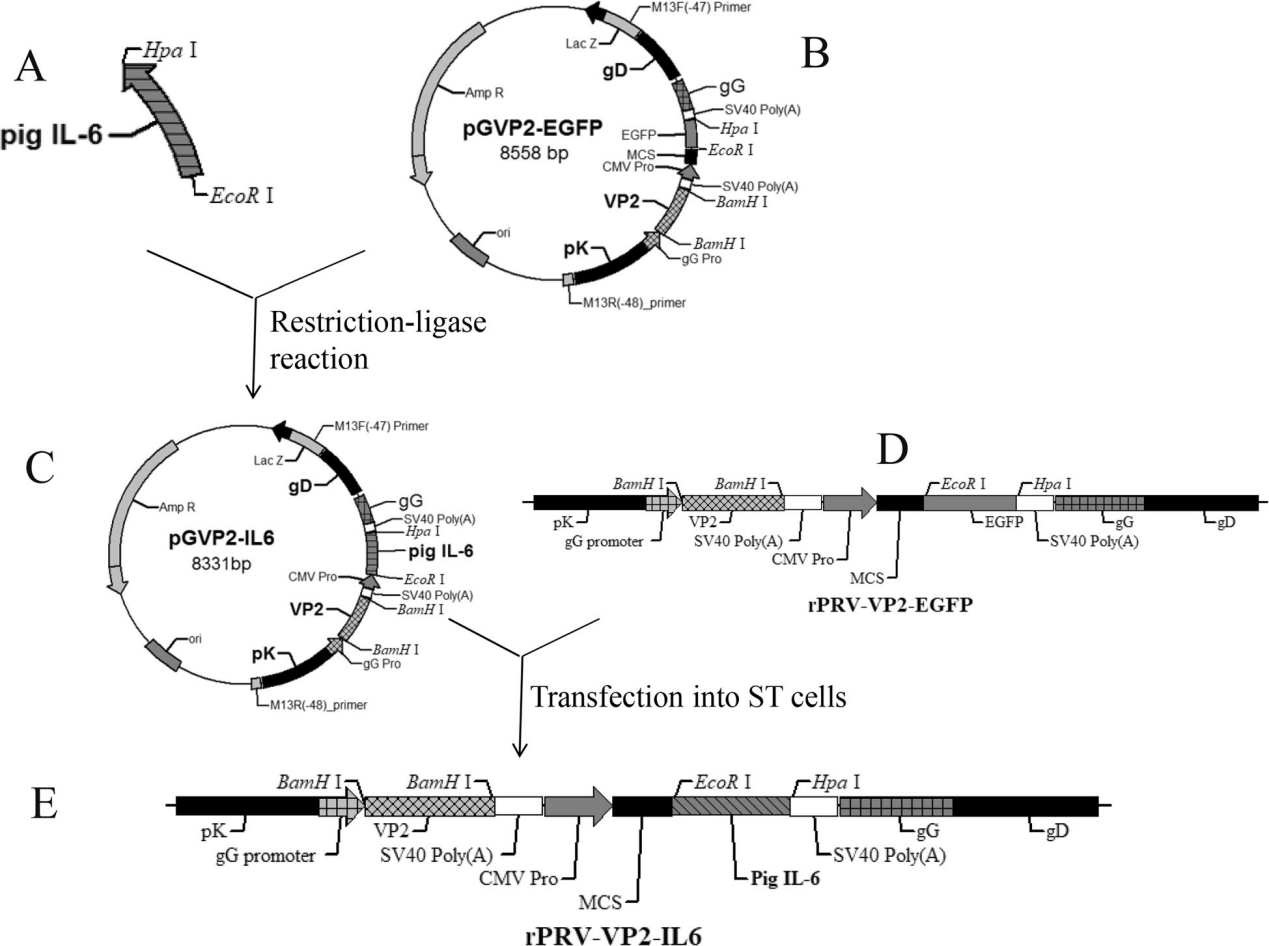
Flowchart of the recombinant pseudorabies virus rPRV-VP2-IL6 expressing porcine parvovirus VP2 protein and porcine IL-6. The recombinant transfer plasmid pGVP2-IL6 was constructed and then transfected into the rPRV-VP2-EGFP-infected ST cells to generate the recombinant virus rPRV-VP2-IL6. **a** The porcine IL-6 digested by *Eco*R I and *Hpa* I. **b** PRV transfer plasmid pGVP2-EGFP containing an *Sph*I–*Kpn*I fragment of the unique short region of PRV strain Fa, SV40 Poly (A), an enhanced GFP (EGFP) expression cassette and the VP2 gene of PPV. **c** The PRV transfer plasmid pGVP2-IL6. The porcine IL-6 was cloned into *Eco*R I- and *Hpa* I-digested the plasmid pGVP2-EGFP to produce plasmid pGVP2-IL6. **d** The genome of rPRV-VP2-EGFP containing the DNA of parental PRV HB98, the *Sph*I–*Kpn*I fragment of the unique short region of PRV strain Fa, SV40 Poly (A), an EGFP expression cassette and the VP2 gene of PPV. **e** The genome of the recombinant virus rPRV-VP2-IL6. The plasmid pGVP2-EGFP was transfected into the rPRV-VP2-EGFP-infected ST cells to generate rPRV-VP2-IL6 containing *Sph*I–*Kpn*I fragment of the unique short region of PRV strain Fa, SV40 Poly (A), pig IL-6 and the VP2 gene of PPV

PRV HB98 with three important virulence factors (TK, gG, and gE) deleted was provided by our laboratory. And PRV NY strain (GenBank accession no. KF130883) isolated in 2012 from piglets with neurological symptoms was used for virus challenge. The PRV HB98 and NY strains were propagated in ST cells. Virulent PPV HN strain was originally isolated in 2013 from the fetuses that spontaneously aborted before 70 days of gestation, which was propagated in ST cells and used for virus challenge. Recombinant plasmid pIRES-IL6 was constructed previously [31]. The PRV transfer plasmid pGVP2-EGFP and the virus rPRV-VP2-EGFP were constructed in our laboratory [33]. The procedures which the plasmid pGVP2-EGFP was constructed were as following: (1) the recombinant plasmid pUP was created by inserting pK, gD and gG gene of PRV-Fa into the restriction sites from *Sph* I to *Kpn* I of the pUC19 vector; (2) the amplified pcDNA3.1(+) SV40 polyA was cloned into pUP with the same enzymes of *BamH* I and *Pst* I to construct the plasmid pUPS; (3) a 1660 bp fragment from the pEGFP-N1 vector was amplified by polymerase chain reaction (PCR), excised by *Pst* I digestion, and then cloned into pUPS restricted with *Pst* I after pUPS had been restricted with *Hind* III and blunted with the klenow fragment of DNA polymerase to generate the final intermediate plasmid pG; (4) the complete VP2 gene from PPV was amplified and ligated into the vector pMD 18-T to generate the recombinant plasmid pMD-VP2; (5) VP2 gene was obtained and ligated into the plasmid pG by digesting each other with *BamH* I, and the recombinant plasmid pGVP2-EGFP (Fig. 1b) was constructed containing an *Sph* I–*Kpn* I fragment of the unique short region of PRV strain Fa, SV40 Poly (A), an enhanced GFP (EGFP) expression cassette and the VP2 gene of PPV. The virus rPRV-VP2-EGFP (Fig. 1d) was generated by transfecting plasmid pGVP2-EGFP and DNA of parental PRV HB98 into ST cells, and purified by selecting green fluorescent plaque purification.

All experimental procedures were reviewed and approved by the Henan Agriculture University Animal Care and Use Committee (license number SCXK (Henan) 2013–0001).

### Construction of the recombinant transfer plasmid

The recombinant transfer plasmid pGVP2-IL6 was constructed as described previously [34]. Briefly, porcine IL-6 gene was amplified by PCR from the plasmid pIRES-IL6 using the specific primers IL6fs and IL6rs (Table 1). The PCR product (Fig. 1a) was digested by *Eco*R I and *Hpa* I, and cloned into similarly digested PRV transfer plasmid pGVP2-EGFP, which EGFP gene was replaced with pig IL-6, driven by the promoter of CMV (pCMV). The resulting plasmid, pGVP2-IL6 (Fig. 1c), was transformed into *Escherichia coli* DH5α, and identified by restriction enzyme digestion and sequencing.

**Table 1:** Primers used for PCR amplification of target genes in this study

Target gene	Primer	Sequence ^a^(5′-3′)	Primer location^b^	Restriction site	Expected product (bp)
Porcine IL-6	IL6fs	GACGAATTCATGAACTCCCTCTCCACA	1–18	*Eco*R I	639
	IL6rs	GTCGTTAAC CTACATTATCCGAATG	471–488	*Hpa* I	
PPV VP2	VP2fs	ACCATGGGGGGGGTTGGTG	2739–2758		1635
	VP2rs	ATCCTATTTCTAGTATAATTT	4359–4373		
PRV gH	gH fs	GCGTGTACTGCGACTGCGTGTT	62,700–62,721		355
	gH rs	CGACCTGGCGTTTATTAACCGAGA	63,032–63,055		
PPV NS1	NS1fs	CCAAAAATGCAAACCCCAATA	1808–1828		194
	NS1rs	TCTGGCGGTGTTGGAGTTAAG	1981–2001		

^a^The restriction enzyme sites used for the construction are underlined

^b^The primers IL6fs/IL6rs were designed to amplify porcine IL-6 gene using plasmid pIRES-IL6 as templates. The primers VP2fs/VP2rs and NS1fs/NS1rs were designed based on the nucleotide sequence (KF429255) of PPV HN genome to amplify VP2 and NS1genes, respectively. The primers gH fs/gH rs were designed to amplify gH gene based on PRV gH nucleotide sequence (KP257591) retrieved from the GenBank

### Homologous recombination and screening of the recombinant virus

The recombinant PRV (rPRV) was generated by homologous recombination. Firstly, rPRV-VP2-EGFP strain at multiplicity of infection (MOI) of 0.01 was inoculated into the monolayer of ST cells of 80% confluency in six-well plates. Three hours later, plasmid pGVP2-IL6 was transfected into PRV-infected ST cells, using 10 μL Lipofectin Reagent 2000 (Invitrogen, Grand Island, NY, USA) for homologous recombination to produce the recombinant virus rPRV-VP2-IL6. Parental PRV-infected ST cells were used as an infection control. The infected cells containing the recombinant virus were collected after the cytopathic effect (CPE) appeared. Subsequently, recombinant viruses were purified by screening plaques with absence of green fluorescence visualized using a standard FITC filter-equipped fluorescence microscope (Nikon Eclipse TS100) in six-well plates and amplified in ST cells for at least three times plaque purification. Gene recombination was further identified by PCR and DNA sequencing, and the expression of the VP2 protein and porcine IL-6 was analyzed by reverse transcription-PCR (RT-PCR) and Western blot.

### Identification of the recombinant virus

#### PCR and RT-PCR

ST cells were harvested at 48 h post-infection (hpi), and then genomic DNA and total RNA were extracted from rPRV-infected ST cells using SDS-proteinase K-phenol and TRIZOL reagent (Gibco BRL), respectively. cDNA was synthesized from the total RNA using Moloney murine leukemia virus (MMLV) reverse transcriptase (Invitrogen Life Technologies, Carlsbad, CA, USA). PCR was amplified using Takara Ex *Taq* DNA polymerase and the primers VP2fs/VP2rs and IL6fs/IL6rs (Table 1) for the VP2 gene and IL-6.

#### Western blot

The expression of VP2 protein and porcine IL-6 in the ST cells infected with rPRV-VP2-IL6 was confirmed by Western blot as previously described [35]. Briefly, ST cell Lysates infected with the virus rPRV-VP2-IL6 were subjected to sodium dodecyl sulfate polyacrylamide gel electropheresis (SDS-PAGE) using 15% gel and then transferred onto a nitrocellulose membrane (Solarbio, Beijing, China). After the membrane was blocked (blocking reagents, Solarbio, Beijing, China), the blots were probed using host rabbit anti-PPV VP2 antibody (biorbyt) or rabbit anti-porcine IL-6 antibody (produced by our laboratory, unpublished data) and developed with horseradish peroxidase (HRP)-labeled anti-rabbit IgG antibody (Invitrogen Life Technologies, Carlsbad, CA, USA). Finally, the substrate, 3, 3-, 5-, 5-tetramethylbenzidine (Promega, New Jersey, USA) was added to corresponding reaction for colour development.

### Growth properties in cell culture

#### Comparison of plaque sizes

The rPRV-VP2-IL6 and parental virus PRV HB98 were inoculated at 200 plaque forming unit (PFU) into ST cells in a six-well plate, respectively. After an absorption period of 2 h at 37 °C, the cells of each well were overlaid with 2 mL 1 × DMEM containing 1.3% low melting point agarose. After virus samples were incubated at 37 °C with 5% CO_2_ for 48 h, the morphogenesis and sizes of the virus plaques were observed using an inverted fluorescence microscope (TS100, Japan).

#### Single-step growth curve analysis

Single-step growth curve was determined as described previously [36]. ST cell monolayers in a number of dishes were infected with either virus rPRV-VP2-IL6 or HB98 at a MOI of 1 per cell. Virus was allowed to absorb cells for 1 h at 37 °C, the inoculums were removed by aspiration, and the cells were washed three times with PBS (0.1 mol/L). The infected cells were over-laid with fresh medium, and harvested at 0, 2, 4, 8, 12, 24, 36, 48, 60 and 72 hpi for titration. Intracellular viruses were released from the infected cells by three freeze-thaw cycles and extracellular virus was cleared of cells and debris by low-speed centrifugation followed by virus titration.

#### Viral growth in different cell types

In vitro growths of rPRV-VP2-IL6 and parental PRV HB98 were determined in ST, Vero, PK-15, IPEC and IBRS2 cells. The ST, Vero, PK-15, IPEC and IBRS2 cells were infected with the viral strains at 0.005 MOI in each well of a six-well plate. After an absorption period of 2 h at 37 °C, the cells were washed with PBS, covered with fresh medium, and then incubated at 37 °C. Cell morphogenesis and CPE without green fluorescence were observed using an inverted fluorescence microscope (TS100, Japan) at 0, 8, 12, 24, 36 and 48 hpi.

The rPRV-VP2-IL6 infectivity was determined by tissue culture infectious dose 50% assay (TCID_50_) in ST, Vero, PK-15, IPEC and IBRS2 cells. The rPRV-VP2-IL6 was serially diluted ten-fold in DMEM in sterile Eppendorf tubes, and 100 μL of the virus dilutions were added to cells in 96-well plates. The plates were examined daily for a discernible CPE, and the final reading was made after 7 days. The virus titer was calculated according to Reed and Muench method [37]. Control wells containing uninoculated cell cultures were included in the test. The virus concentration was expressed as TCID_50_/mL.

### Determination of chemical and physical properties of the rPRV-VP2-IL6

The procedures to determine chemical and physical properties of the virus were performed as described previously [38]. Chemical and physical properties included testing resistance to chloroform and trypsin, sensitivity to pH, and stability to UV irradiation treatment for PRV. The recombinant virus was treated by chloroform for 10 min, 0.25% trypsin for 10 min, 5% carbolic acid for 2 min, UV irradiation for 30 min, and 1% sodium hydroxide for 10 min, respectively. 100 μL of each treated sample was inoculated into ST cells, and the CPE was examined daily. Recombinant virus without any treatment was used as a positive CPE control.

### Genetic stability

The rPRV-VP2-IL6 was serially passaged to the 15th passage on ST cells. Infected cells without green fluorescence were observed, and collected when the CPE was 80%. After freeze-thawing three times, DNA was extracted from infected cells from passage 5, 10 and 15. The PPV VP2 gene and porcine IL-6 were amplified using Takara Ex *Taq* DNA polymerase and the primer sets as described above, and sequenced from the passages, respectively.

### Immunization of mice with rPRV

A total of 125 six-week-old female Balb/c mice were randomly divided into five groups of 25. Three groups were vaccinated with 5log10 TCID_50_ of rPRV-VP2-IL6, rPRV-VP2-EGFP or PRV HB98. As controls, the other two groups were injected with 100 μL of commercial PPV inactivated vaccine and 100 μL of DMEM (unvaccinated control), respectively. All mice were injected by the intramuscular (IM) route and boosted with the same dose and route 4 weeks later. Blood samples from five mice in each group were collected via the tail vein on weeks 0, 2, 4, 6 and 8 after the initial immunization for immunoassays.

#### Serum neutralization assay for PRV

Serum neutralizing antibodies against PRV were detected using a microtiter virus neutralization test. Briefly, sera were inactivated at 56 °C for 30 min and then two-fold serially diluted. 50 μL of the serially diluted sera and 200 TCID_50_ of the PRV NY strain were mixed together and incubated at 37 °C with 5% CO_2_ for 1 h, and then added into ST cells in 96-well flat-bottom plates to incubate for another 4–6 days. Antiserum titers were expressed as the highest dilution that reduced the CPE of PRV NY strain by 50% compared with non-neutralized controls. All samples were analyzed in triplicate, and the results were presented as the mean of the triplicate assays.

#### Hemagglutination inhibition test for PPV

Hemagglutination inhibition assay for PPV was performed as described previously [39]. Briefly, 100 μL mouse sera were heat-inactivated at 56 °C water bath for 30 min, and then treated with 300 μL of 25% kaolin suspension at room temperature for 30 min. The supernatant was absorbed with 100 μL 25% guinea pig red blood cells (RBCs) at 37 °C for 1 h. Final supernatant was regarded as a 1:4 dilution of the original serum.

50 μL PBS solution was added from the second well to the 12th well in the 96-well V-bottom microplates; 50 μL of the treated sera were added into the 1st and 2nd wells of each row respectively, diluted from the second well to the 10th well, and then 50 μL of them were discard, while the 11th and 12th wells were used as virus control and red blood cell control; To each well was added 50 μL of 4 HA units of antigen. After incubating at 37 °C for 1 h, 50 μL of 0.6% guinea pig RBCs were added. The contents were mixed, incubated at room temperature for 2 h, and examined for inhibition of HA. That 50% of RBCs showed agglutination inhibition was used as the end point, and HI titer was defined as the reciprocal of the highest dilution inhibiting HA.

#### ELISA for antibodies against PPV

The sera were used to determine the titer of PPV-specific antibodies by ELISA (Wuhankeqian Animal Biological Products Co., Ltd.). Serum samples from mice were diluted to 1:40, and 100 μL was added to 96-well ELISA plates per well. 100 μL of HRP-conjugated goat anti-mouse IgG (Sigma Chemical Co., St. Louis, MO, USA), diluted to 1:5000, was added into each well to detect bound mouse antibodies. 100 μL of TMB (3, 3′, 5, 5′-tetramethyl benzidine) was added, and the plates were incubated for 10 min in the dark before the color was developed. The OD was measured on an ELISA reader at 630 nm.

#### PPV-specific lymphocyte proliferation response assay

At 8 weeks post-initial immunization, five mice in each group were sacrificed for lymphocyte proliferation assay (LPA). PPV-specific LPA was performed using splenic mononuclear cells (SMCs) of immunized mice, as described previously [34]. Briefly, spleens were collected aseptically from the immunized mice and homogenized in PBS (pH 7.4). SMCs were prepared using Histopaque-1077 (Sigma) and plated in 96-well flat-bottom plates at 100 μL/well (2 × 10^5^ cells/well) in triplicate. Subsequently, one sample was treated with 100 TCID_50_/mL PPV, one with 5 μg/mL Concanavalin A (Sigma) as a positive control, and one with medium alone as a negative control. After incubation at 37 °C in 5% CO_2_ for 60 h, a 20-μL aliquot of CellTiter 96 Aqueous One Solution Reagent (Promega, Madison, WI, USA) was added into each well according to the manufacturer’s protocol. After a 4-h incubation at 37 °C, the absorbance was read at 490 nm. Finally, the plates were read at OD_490_ nm and the stimulation index (SI) was calculated as the ratio of the average OD value of wells containing antigen-stimulated cells to the average OD value of wells containing cells without antigen.

#### Challenge experiments

Eight weeks after the first immunization, the remaining 20 mice of each group were divided into two subgroups of 10 and challenged with PRV NY or PPV HN. The mice were challenged with 0.5 mL (6log10 TCID_50_/mL) by subcutaneous injection. The mice challenged with PRV NY were monitored daily for clinical signs, and the number surviving was recorded for 2 weeks. A pair of primers gHfs and gHrs (Table 1) was designed based on a highly conserved sequence within the gH region of PRV genome. PRV nucleic acid in lung samples from dead mice was detected by PCR using the specific primers gHfs and gHrs.

To evaluate the level of protection in response to the challenge, PPV DNA was detected by PCR amplification of the sequence encoding for non-structural protein NS1 from tissue samples of PPV challenged mice. Mice challenged with PPV HN were euthanized at 14 dpi. The hearts, livers, lungs, spleens and kidneys were harvested for detection of PPV nucleic acid by PCR using specific primers NS1fs and NS1rs (Table 1) based on the PPV NS1 gene of NADL-2 strain (Gen Bank no.NC 001718). Total protection was defined as the absence of detectable virus.

### Statistical analysis

As to the analysis of the data, normality in the repeated measures and homogeneity of variance were tested with Shapiro Wilk Test and Levene’s test, respectively. Differences between groups were analyzed by an analysis of variance (ANOVA) and a Student’s t-test. *P*-values less than 0.05 were defined as significant, and those less than 0.01 were regarded as highly significant.

## Results

### Construction of a recombinant pseudorabies virus

A transfer plasmid pGVP2-IL6 (Fig. 1c) was constructed by inserting the porcine IL-6 gene (639 bp) under the control of CMV gene promoter and substituting for EGFP gene of pGVP2-EGFP, and transfected into ST cells pre-infected with the rPRV-VP2-EGFP strain. The resulting recombinant PRV, rPRV-VP2-IL6 (Fig. 1e), was generated by inserting porcine IL-6 gene into the PRV genome via homologous recombination.

ST cells infected with rPRV-VP2-IL6 showed no green fluorescence under fluorescence microscopy. Obvious CPEs were observed approximately 36 h after transfection, and then rPRV-VP2-IL6 was harvested. Recombinant viruses were purified by green fluorescent plaque-formation assay until all clones showed no green fluorescence. After three rounds of plaque purification, all clones were the recombinant viruses. The porcine IL-6 gene in the rPRV-VP2-IL6 was detected by PCR, and was identical to the predicted DNA fragment size (639 bp), but was not present in the parental virus (data not shown). The sequence analysis showed IL-6 gene sequence was 100% identical with the porcine IL-6 of plasmid pIRES-IL6. This verified that porcine IL-6 was correctly inserted into the downstream region of the CMV gene promoter.

### Expression of VP2 and porcine IL-6 proteins in the recombinant virus

To confirm the expression of VP2 protein and porcine IL-6 in ST cells, total RNA was extracted from the ST cells infected with rPRV-VP2-IL6 after 48 h and analyzed by RT-PCR. 1.6 kb in size for the VP2 gene and 0.6 kb for the porcine IL-6 gene of the extracted RNA samples were cloned and sequenced. The results showed the nucleotide sequences of VP2 gene and porcine IL-6 were identical to VP2 (KF429255) and the porcine IL-6 of plasmid pIRES-IL6, respectively.

**Fig. 2:**
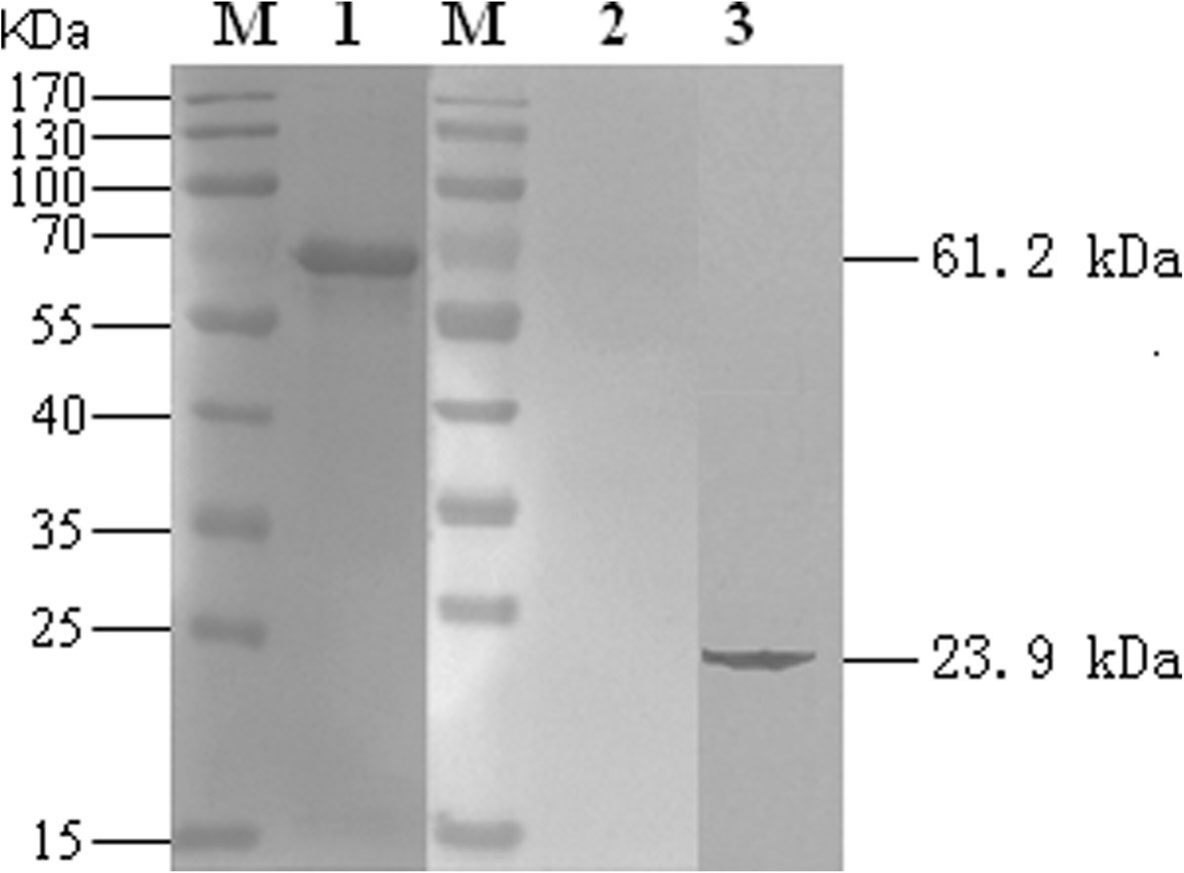
Western blot analysis of total lysates of ST cells transfected with rPRV-VP2-IL6 (lane 1), parental PRV HB98 (lane 2) and rPRV-VP2-IL6 (lane 3), respectively, using host rabbit anti-PPV VP2 antibody or using rabbit anti-porcine IL-6 antibody

As shown in Fig. 2, the lysates from the ST cells infected with rPRV-VP2-IL6 contained a protein with a molecular weight of 61.2 kDa (VP2 protein) (Fig. 2, Lane 1) and a protein with a weight of 23.9 kDa (porcine IL-6 protein) (Fig. 2, Lane 3). No bands were observed in ST cells with PRV HB98 (Fig. 2, Lane 2). Thus, the VP2 protein of PPV and porcine IL-6 protein could be expressed correctly in the recombinant rPRV-VP2-IL6.

### The growth properties of the virus

The morphology of ST cells was normal at 12 hpi and then shrunk at 24 hpi. Many cells were observed obvious shedding at 36 hpi and the shed cells increased to 85% at 48 hpi. There were no obvious differences in the morphogenesis and size of virus plaques between rPRV-VP2-IL6 and parental strain HB98.

**Fig. 3:**
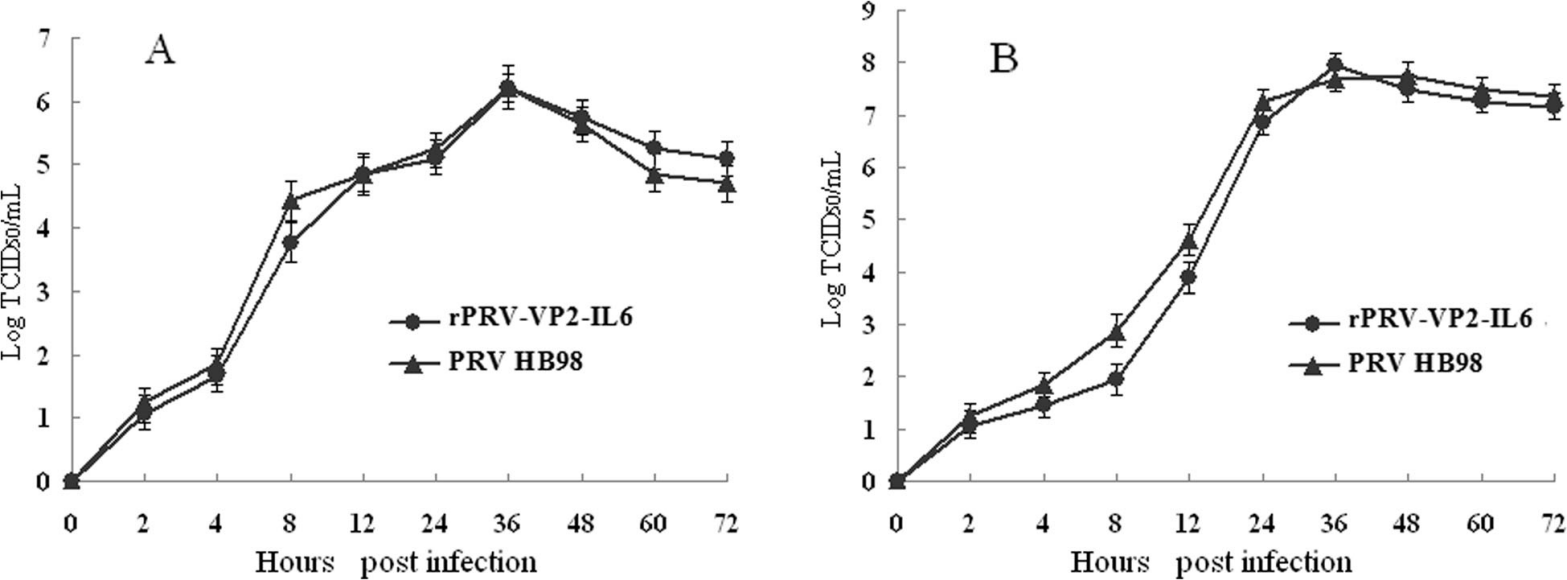
Single-step growth curve analysis of the recombinant rPRV-VP2-IL6. The recombinant rPRV-VP2-IL2 () and parental PRV HB98 () were infected ST cells at an MOI of 200 PFU. Plates were harvested at indicated time points post-infection, and separated into cell-free medium and cells as described in Materials and Methods. Plaque-forming virus titers were determined post-infection. **a**: Extrcellular viruses; **b**: Intracellular viruses

Single-step growth curve analysis was used to determine the rates of intracellular and extracellular infectious virus formation, and to verify that the expression of foreign VP2 gene did not adversely affect viral growth. After infection of ST cells with rPRV-VP2-IL6 or PRV HB98, the cell and supernatant fractions were harvested at various times. The titers of extracellular and intracellular viruses were determined on ST cells and used to plot single-step growth curves. No obvious difference was shown between the two viruses, and high virus titers (>6log10/mL TCID_50_) were detected in both viruses (Fig. 3).

The rPRV-VP2-IL6 could form clear plaques on ST, PK-15, IBRS-2, Vero, and IPEC cells, and the plaques appeared at the same time. In addition, the TCID_50_ of the recombinant virus on ST, PK-15, IBRS-2, Vero, and IPEC cells were 6.625log10/mL, 4.125log10/mL, 6.25log10/mL, 4.625log10/mL, and 6log10/mL, respectively, whereas the parental strain had 6.25log10/mL, 5.125log10/mL, 6.75log10/mL, 5log10/mL, and 6.125log10/mL TCID_50_ for ST, PK-15, IBRS-2, Vero, and IPEC cells, respectively. The results showed that proliferation titer of the recombinant virus was similar to the parental strain on the ST, IBRS-2 and IPEC cells, suggesting these cells were more suitable for culture of PRV.

### The physicochemical and genetic stability properties of the virus

The results of physicochemical characters on the recombinant virus indicated that the rPRV-VP2-IL6 was sensitive to chloroform, 0.25% trypsase, 5% carbolic acid, ultraviolet rays irradiating (30 min) and 1% sodium hydroxide.

After 15 generations of monoclonal recombinant virus, no green fluorescence was observed in ST cells. 10 plaques were randomly picked and used to amplify the VP2 gene and porcine IL-6 by PCR. Both the 1635 bp-fragment of VP2 gene and the 639 bp-fragment of porcine IL-6 gene were amplified in all 10 plaques. The sequences of the 1635 bp and 639 bp PCR products were same as the sequence of PPV HN (KF429255) and the porcine IL-6 of plasmid pIRES-IL6, respectively, showing that the recombinant virus carrying the VP2 gene and porcine IL-6 had genetic stability.

### Neutralizing antibodies against PRV

Neutralizing antibodies were detected as early as 2 weeks after a single immunization in the sera of mice immunized with rPRV or HB98. Moreover, all mice immunized with rPRV and HB98 developed neutralizing antibodies with titers of 1:8 at week 4 post-first vaccination and 1:64–1:128 at week 4 post-second vaccination (Table 2). No neutralizing antibodies against PRV were detected in the mice injected with PPV vaccine and DMEM.

**Table 2:** PRV-specific neutralizing antibody (NA) and PPV-specific HI antibody (Log _2_)^a^ levels in mice

Group	Weeks post the first vaccination									
	0		2		4		6		8	
	NA	HI	NA	HI	NA	HI	NA	HI	NA	HI
rPRV-VP2-IL6	-^b^	0	1:2	2.5 ± 0.2^* c^	1:8	4.1 ± 0.3^*^	1:32	5.3 ± 0.2^*^	1:128	6.9 ± 0.3^*^
rPRV-VP2-EGFP	–	0	1:2	2.0 ± 0.2^*^	1:8	3.9 ± 0.2^*^	1:32	5.0 ± 0.3^*^	1:64	6.5 ± 0.2^*^
PPV vaccine	–	0	–	4.0 ± 0.3^*^	–	6.3 ± 0.3^*^	–	5.6 ± 0.2^*^	–	7.6 ± 0.3^*^
PRV HB98	–	0	1:2	0	1:8	0	1:32	0	1:128	0
DMEM	–	0	–	0	–	0	–	0	–	0

^a^Values are expressed as mean HI antibody titer ± standard error

^b^NA titers <2

^c^Statistically significant differences (*P* < 0.05) are indicated by*(compared with compared with PRV HB98 or DMEM)

### PPV specific antibodies

To evaluate PPV specific antibody responses, antigen-specific antibodies were detected using HI and an indirect ELISA. As shown in Table 2, HI antibodies were detected with the lower level 2 weeks later after vaccination with rPRV. No detectable HI antibody was elicited from mice vaccinated with either PRV HB98 or DMEM before or after immunization, whereas HI antibody titers reached above 6 log2 at 8 weeks after vaccination with rPRV (*P* < 0.05). However, rPRV-VP2-IL6 group evoked slightly higher HI antibody level than rPRV-VP2-EGFP, but not significantly different (*P* > 0.05).

**Fig. 4:**
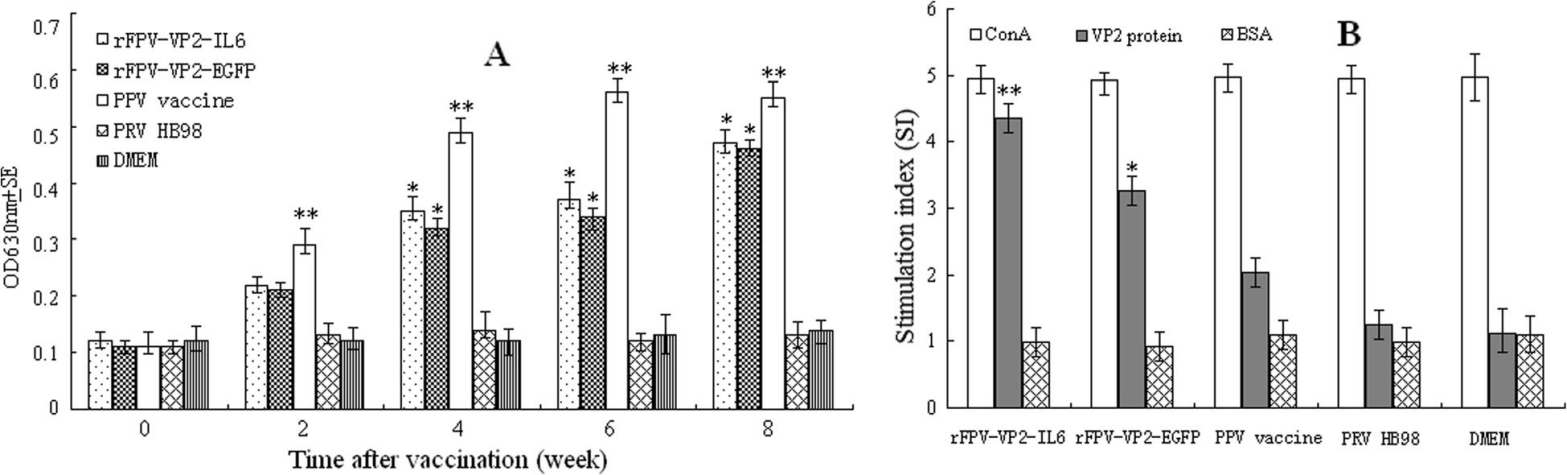
Immunogenicity of rPRV-VP2-IL6 in mice. Five groups of 25 Balb/c mice received injection of 100 μL of solution containing either with 5log10 TCID_50_ of rPRV-VP2-IL6, rPRV-VP2-EGFP, PRV HB98, PPV vaccine or DMEM at both week 0 and week 4. Sera were collected on weeks 0, 2, 4, 6, 8 after the initial immunization and 5 mice were sacrificed at week 8 for lymphocyte proliferation assay. **a** Anti- PPV antibodies determined by ELISA. **b** PPV specific lymphocyte proliferation assay. Values are expressed as mean counts ±standard error. Statistically significant differences (*P* < 0.05) are indicated by * or **

PPV-specific ELISA antibody titers reached detectable levels in mice immunized with PPV vaccine or rPRV 2 weeks after the initial injection, and the antibody levels were further increased subsequently (Fig. 4a). Higher total levels of PPV Ag specific antibodies were induced by rPRV-VP2-IL6 compared with those induced by rPRV-VP2-EGFP although this difference did not reach the level of statistical significance (*P* > 0.05). No antibody against PPV was detected over the 8 weeks in the groups vaccinated with parental PRV HB98 or DMEM.

### PPV specific lymphocyte proliferation response

At 8 weeks post-initial vaccination, the SMCs from mice immunized with rPRV-VP2-IL6, rPRV-VP2-EGFP, PPV vaccine, PRV HB98 or medium were prepared to assess the proliferative immune responses to PPV. SMCs from the rPRVs and PPV vaccine-vaccinated groups showed the increased proliferative response to PPV when stimulated with PPV, whereas the mice vaccinated with the parental PRV HB98 strain or DMEM did not (Fig. 4b). The PPV specific proliferation responses were significantly higher in mice immunized with rPRV-VP2-IL6 compared with those immunized with rPRV-VP2-EGFP (*P* < 0.01).

### Protection of mice against virulent challenge

At 8 weeks post-initial vaccination, 10 mice of each group were challenged with 6log10 TCID_50_ of the virulent PRV NY strain. The mice started to show itching or died from viral infection on day 2 after challenge. In the PPV vaccine or DMEM injected control group, all mice died and developed neurological symptoms of PRV infection before death, and two out of ten mice died in both rPRV-VP2-EGFP- and HB98-immunized groups, whereas one of ten mice injected with rPRV-VP2-IL6 died. PRV DNA was detected in lung samples from the dead mice (data not shown).

The protective efficacy of rPRVs was tested by measuring inhibition of PPV replication in multiple organs. PCR analysis demonstrated that PPV was detected in tissue samples from all mice (10/10) immunized with either the PRV HB98 or DMEM, whereas only one and two mice were positive for the presence of virus in the tissues for the rPRV-VP2-IL6- and rPRV-VP2-EGFP-immunized groups respectively, namely, 9 and 8 mice were protected from PPV challenge, and all mice immunized with PPV vaccine were protected.

## Discussion

In recent years, attenuated PRV has been used successfully as a virus vector to express protective antigens of many pathogens. The recombinant transfer plasmid together with genomic DNA of attenuated PRV strain was usually co-transfected into mammalian cells to generate a rPRV, and different attenuated PRV strains were used as parental strains. PRV Bartha-k61 strain has been used as a parental strain to construct rPRVs expressing PRRSV GP5 [25], H3N2 subtype swine influenza virus HA [40], rabies virus rgp [41], *Schistosoma japonicum* Sj26GST and SjFABP [42] and *Toxoplasma gondii* TgSAG1 gene [43]. Chen et al. constructed a rPRV expressing PPV VP2 gene using PRV strain SA215 as the parental strain [28]. Other studies have reported using a TK^−^/gG^−^/LacZ^+^ mutant of PRV Ea strain to construct rPRVs expressing Japanese encephalitis virus NSl gene [44], foot and mouth disease virus P12A and 3C genes [26]. In this study, PRV HB98, a PRV TK^−^/gG^−^/gE^−^ three-gene deletion strain, was used as the parental strain. PRV HB98 strain is an approved vaccine strain that is strongly immunogenic and has been used widely to prevent and control PRV infection in China. Furthermore, HB98 isolated from China has clear genetic background. Therefore, HB98 strain is a more suitable parental strain to express the PPV VP2 gene for immunization against PPV infection.

The protective efficacy of a rPRV encoding the PPV VP2 in mice and swine was investigated previously [28, 33], However, immune efficacy was not satisfied in vivo. IL-6 is known to mediate antibody production and the regulation of Th1 responses [45], and previous studies showed that IL-6 could enhance the protection against challenge with the infectious agent [31, 46]. Therefore, rPRV-VP2-IL6 containing the PPV VP2 and porcine IL-6 genes was constructed to enhance the immunogenicity of VP2 protein of PPV and assess its efficacy for PRV and PPV. In this study, the properties of rPRV-VP2-IL6 were similar to the parental virus HB98 in vitro including growth properties, growth curves and virus plaque sizes. This showed the insertion of the VP2 gene and porcine IL-6 did not affect the normal replication of PRV, which was coincided with the results reported by Yuan et al. [41]. In addition, the physical and chemical properties indicated that rPRV-VP2-IL6 had properties in common with PRV. Genetic stability and safety tests in mice showed that rPRV-VP2-IL6 had excellent features as a vaccine candidate strain. And taken the biosafety of the vaccine candidate into consideration, the rPRV-VP2-IL6 constructed in this study was free of the foreign EGFP gene.

In this study, the rPRV-immunized groups showed detectable antibody levels of immune response in mice 2 weeks after the initial immunization, and the antibody titer in mice immunized with rPRV-VP2-IL6 was slightly higher than that in mice immunized with rPRV-VP2-EGFP, but not significantly different (*P* > 0.05, Table 2 and Fig. 4a). Our results showed that the T cells of mice immunized with rPRV exhibited a PPV-specific lymphocyte proliferative response, which was significantly higher in mice immunized with rPRV than in mice immunized with the PPV vaccine (*P* < 0.01, Fig. 4b). Thus, the rPRV-VP2-IL6 stimulated a stronger immune response to PPV. Similar results were also reported previously [28].

In terms of the level of protection against PRV elicited by the rPRV-VP2-IL6, the mice immunized with rPRV or HB98 developed PRV-specific neutralizing antibodies at titers 1:64–1:128 at week 4 post-second vaccination (Table 2), there was no significant difference between them. However, one and two mice died in the PRV rPRV-VP2-IL6- and HB98-immunized groups, respectively. Thus, the rPRV-VP2-IL6 or HB98 provided partial protection against challenge with the PRV NY strain. This might reflect antigenic variation between PRV NY strain and HB98, which was consistent with the results for the Bartha-K61 vaccine, which did not provide effective protection against PRV HeN1 or PRV JS-2012 infection [47, 48].

The paper provided evidence that PRV HB98 represents a useful vector for expression of a heterogeneous gene, and suggested that rPRV-VP2-IL6 is a promising vaccine candidate to control PRV and PPV infections. However, piglets still have considerable levels of maternally derived antibodies (MDA), and killed vaccines successfully overcome this MDA. Whether the replicative vaccine will work in pigs and successfully overcome this MDA should be studied in the future.

## Conclusions

rPRV-VP2-IL6 stimulated a stronger immune response to PPV, and could provide partial protection from challenge of the emerging PRV variant, suggesting that rPRV-VP2-IL6 might be a potential candidate vaccine against PRV and PPV infections in pigs.

## Data Availability

All data generated or analyzed during this study are included in this published article and GeneBank.

## Abbreviations

CPE: Cytopathic effect
DMEM: Dulbecco’s modified Eagle medium
EGFP: Enhanced GFP
FBS: Fetal bovine serum
hpi: Hours post-infection
HRP: Horseradish peroxidase
IL-6: Interleukin-6
IM: Intramuscular
kb: Kilobases
LPA: Lymphocyte proliferation assay
m.o.i: Multiplicity of infection
MMLV: Moloney murine leukemia virus
ORFs: Open reading frames
PCR: Polymerase chain reaction
PCV2: Porcine circovirus 2
PPV: Porcine parvovirus
PRRSV: Porcine reproductive and respiratory syndrome virus
PRV: Pseudorabies virus
rPRV: Recombinant PRV
RT-PCR: Reverse transcription-PCR
SDS-PAGE: Sodium dodecyl sulfate polyacrylamide gel electropheresis
SI: Stimulation index
SMCs: Splenic mononuclear cells
ST: Swine testicle
TCID_50_: Tissue culture infectious dose 50% assay

## References

[1] Jeoung HY, Lim SI, Kim JJ, Cho YY, Kim YK, Song JY (2015). Serological prevalence of viral agents that induce reproductive failure in south Korean wild boar. BMC Vet Res.

[2] Kaur A, Mahajan V, Leishangthem GD, Singh ND, Bhat P, Banga HS (2016). Epidemiological and immunopathological studies on porcine parvovirus infection in Punjab. Vet World.

[3] Mengeling WL, Cutlip RC (1976). Reproductive disease experimentally induced by exposing pregnant gilts to porcine parvovirus. Am J Vet Res.

[4] Streck AF, Canal CW, Truyen U (2015). Molecular epidemiology and evolution of porcine parvoviruses. Infect Genet Evol.

[5] Meszaros I, Olasz F, Csagola A, Tijssen P, Zadori Z. Biology of porcine parvovirus (ungulate parvovirus 1). Viruses. 2017;9. 10.3390/v9120393 PMC5744167 29261104

[6] Hu L, Lin X, Nie F, ZexiaoYang, Yao X, Li G (2016). Simultaneous typing of seven porcine pathogens by multiplex PCR with a GeXP analyser. J Virol Methods.

[7] Ouyang T, Zhang X, Liu X, Ren L. Co-Infection of Swine with Porcine Circovirus Type 2 and Other Swine Viruses. Viruses. 2019;11. 10.3390/v11020185 PMC6410029 30795620

[8] Kamstrup S, Langeveld J, Botner A, Nielsen J, Schaaper WM, Boshuizen RS (1998). Mapping the antigenic structure of porcine parvovirus at the level of peptides. Virus Res.

[9] Streck AF, Bonatto SL, Homeier T, Souza CK, Goncalves KR, Gava D (2011). High rate of viral evolution in the capsid protein of porcine parvovirus. J Gen Virol.

[10] Xu YG, Cui LC, Wang HW, Huo GC, Li SL (2013). Characterization of the capsid protein VP2 gene of a virulent strain NE/09 of porcine parvovirus isolated in China. Res Vet Sci.

[11] Ji P, Liu Y, Chen Y, Wang A, Jiang D, Zhao B (2017). Porcine parvovirus capsid protein expressed in Escherichia coli self-assembles into virus-like particles with high immunogenicity in mice and Guinea pigs. Antivir Res.

[12] Pan Q, He K, Wang Y, Wang X, Ouyang W (2013). Influence of minor displacements in loops of the porcine parvovirus VP2 capsid on virus-like particles assembly and the induction of antibody responses. Virus Genes.

[13] Foerster T, Streck AF, Speck S, Selbitz HJ, Lindner T, Truyen U (2016). An inactivated whole-virus porcine parvovirus vaccine protects pigs against disease but does not prevent virus shedding even after homologous virus challenge. J Gen Virol.

[14] Guo C, Zhong Z, Huang Y (2014). Production and immunogenicity of VP2 protein of porcine parvovirus expressed in Pichia pastoris. Arch Virol.

[15] Tian W, Qiu Z, Cui Y, Zhang J, Ma X, Cui S (2019). Comparison of immune responses induced by porcine parvovirus virus-like particles and inactivated vaccine. Pak J Pharm Sci.

[16] Davison AJ, Eberle R, Ehlers B, Hayward GS, McGeoch DJ, Minson AC (2009). The order Herpesvirales. Arch Virol.

[17] Klupp BG, Hengartner CJ, Mettenleiter TC, Enquist LW (2004). Complete, annotated sequence of the pseudorabies virus genome. J Virol.

[18] Olsen LM, Ch'ng TH, Card JP, Enquist LW (2006). Role of pseudorabies virus Us3 protein kinase during neuronal infection. J Virol.

[19] Kimman TG, de Wind N, Oei-Lie N, Pol JM, Berns AJ, Gielkens AL (1992). Contribution of single genes within the unique short region of Aujeszky's disease virus (suid herpesvirus type 1) to virulence, pathogenesis and immunogenicity. J Gen Virol.

[20] Sun Y, Luo Y, Wang CH, Yuan J, Li N, Song K (2016). Control of swine pseudorabies in China: opportunities and limitations. Vet Microbiol.

[21] Zhou J, Li S, Wang X, Zou M, Gao S (2017). Bartha-k61 vaccine protects growing pigs against challenge with an emerging variant pseudorabies virus. Vaccine..

[22] Freuling CM, Muller TF, Mettenleiter TC (2017). Vaccines against pseudorabies virus (PrV). Vet Microbiol.

[23] Lei JL, Xia SL, Wang Y, Du M, Xiang GT, Cong X (2016). Safety and immunogenicity of a gE/gI/TK gene-deleted pseudorabies virus variant expressing the E2 protein of classical swine fever virus in pigs. Immunol Lett.

[24] Qian P, Zhi X, Wang B, Zhang H, Chen H, Li X (2015). Construction and immune efficacy of recombinant pseudorabies virus expressing PrM-E proteins of Japanese encephalitis virus genotype capital I. Ukrainian Virol J.

[25] Qiu HJ, Tian ZJ, Tong GZ, Zhou YJ, Ni JQ, Luo YZ (2005). Protective immunity induced by a recombinant pseudorabies virus expressing the GP5 of porcine reproductive and respiratory syndrome virus in piglets. Vet Immunol Immunopathol.

[26] Zhang K, Huang J, Wang Q, He Y, Xu Z, Xiang M (2011). Recombinant pseudorabies virus expressing P12A and 3C of FMDV can partially protect piglets against FMDV challenge. Res Vet Sci.

[27] Lehmann D, Sodoyer R, Leterme S, Crevat D (2002). Improvement of serological discrimination between herpesvirus-infected animals and animals vaccinated with marker vaccines. Vet Microbiol.

[28] Chen Y, Guo W, Xu Z, Yan Q, Luo Y, Shi Q (2011). A novel recombinant pseudorabies virus expressing parvovirus VP2 gene: immunogenicity and protective efficacy in swine. Virol J.

[29] Tanaka T, Narazaki M, Masuda K, Kishimoto T (2016). Regulation of IL-6 in immunity and diseases. Adv Exp Med Biol.

[30] Su B, Wang J, Wang X, Jin H, Zhao G, Ding Z (2008). The effects of IL-6 and TNF-alpha as molecular adjuvants on immune responses to FMDV and maturation of dendritic cells by DNA vaccination. Vaccine..

[31] Guo XQ, Wang LQ, Qiao H, Yang XW, Yang MF, Chen HY (2015). Enhancement of the immunogenicity of a porcine circovirus type 2 DNA vaccine by a recombinant plasmid coexpressing capsid protein and porcine interleukin-6 in mice. Microbiol Immunol.

[32] Jiang W, Jin N, Cui S, Li Z, Zhang L, Wang H (2006). Enhancing immune responses against HIV-1 DNA vaccine by coinoculating IL-6 expression vector. J Virol Methods.

[33] Fu P, Pan X, Han Q, Yang X, Zhu Q, Guo X (2016). Immune response of recombinant Pseudorabies virus rPRV-VP2 expressing VP2 gene of porcine parvovirus in mice. Bing Du Xue Bao.

[34] Zheng LL, Guo XQ, Zhu QL, Chao AJ, Fu PF, Wei ZY (2015). Construction and immunogenicity of a recombinant pseudorabies virus co-expressing porcine circovirus type 2 capsid protein and interleukin 18. Virus Res.

[35] Pan Q, Wang H, Ouyang W, Wang X, Bi Z, Xia X (2016). Immunogenicity of adenovirus-derived porcine parvovirus-like particles displaying B and T cell epitopes of foot-and-mouth disease. Vaccine..

[36] Trapp S, Osterrieder N, Keil GM, Beer M (2003). Mutagenesis of a bovine herpesvirus type 1 genome cloned as an infectious bacterial artificial chromosome: analysis of glycoprotein E and G double deletion mutants. J Gen Virol.

[37] Zhao FR, Xie YL, Liu ZZ, Shao JJ, Li SF, Zhang YG (2017). Lithium chloride inhibits early stages of foot-and-mouth disease virus (FMDV) replication in vitro. J Med Virol.

[38] Binn LN, Lazar EC, Eddy GA, Kajima M (1970). Recovery and characterization of a minute virus of canines. Infect Immun.

[39] Joo HS, Donaldson-Wood CR, Johnson RH (1976). A standardised haemagglutination inhibition test for porcine parvovirus antibody. Aust Vet J.

[40] Tian ZJ, Zhou GH, Zheng BL, Qiu HJ, Ni JQ, Yang HL (2006). A recombinant pseudorabies virus encoding the HA gene from H3N2 subtype swine influenza virus protects mice from virulent challenge. Vet Immunol Immunopathol.

[41] Yuan Z, Zhang S, Liu Y, Zhang F, Fooks AR, Li Q (2008). A recombinant pseudorabies virus expressing rabies virus glycoprotein: safety and immunogenicity in dogs. Vaccine..

[42] Wei F, Zhai Y, Jin H, Shang L, Men J, Lin J (2010). Development and immunogenicity of a recombinant pseudorabies virus expressing Sj26GST and SjFABP from Schistosoma japonicum. Vaccine..

[43] Shang L, Liu Q, Liu W, Men J, Gao S, Jiang L (2009). Protection in mice immunized with a heterologous prime-boost regime using DNA and recombinant pseudorabies expressing TgSAG1 against toxoplasma gondii challenge. Vaccine..

[44] Xu G, Xu X, Li Z, He Q, Wu B, Sun S (2004). Construction of recombinant pseudorabies virus expressing NS1 protein of Japanese encephalitis (SA14-14-2) virus and its safety and immunogenicity. Vaccine..

[45] Kishimoto T (2006). Interleukin-6: discovery of a pleiotropic cytokine. Arthritis Res Ther.

[46] Wang G, Pan L, Zhang Y, Wang Y, Zhang Z, Lu J (2011). Intranasal delivery of cationic PLGA nano/microparticles-loaded FMDV DNA vaccine encoding IL-6 elicited protective immunity against FMDV challenge. PLoS One.

[47] An TQ, Peng JM, Tian ZJ, Zhao HY, Li N, Liu YM (2013). Pseudorabies virus variant in Bartha-K61-vaccinated pigs, China, 2012. Emerg Infect Dis.

[48] Yu ZQ, Tong W, Zheng H, Li LW, Li GX, Gao F (2017). Variations in glycoprotein B contribute to immunogenic difference between PRV variant JS-2012 and Bartha-K61. Vet Microbiol.

